# Improving Children’s Knowledge of Fraction Magnitudes

**DOI:** 10.1371/journal.pone.0165243

**Published:** 2016-10-21

**Authors:** Lisa K. Fazio, Casey A. Kennedy, Robert S. Siegler

**Affiliations:** 1 Department of Psychology, Carnegie Mellon University, Pittsburgh, PA, United States of America; 2 The Siegler Center for Innovative Learning, Beijing Normal University, Beijing, China; Katholieke Universiteit Leuven, BELGIUM

## Abstract

We examined whether playing a computerized fraction game, based on the integrated theory of numerical development and on the Common Core State Standards’ suggestions for teaching fractions, would improve children’s fraction magnitude understanding. Fourth and fifth-graders were given brief instruction about unit fractions and played *Catch the Monster with Fractions*, a game in which they estimated fraction locations on a number line and received feedback on the accuracy of their estimates. The intervention lasted less than 15 minutes. In our initial study, children showed large gains from pretest to posttest in their fraction number line estimates, magnitude comparisons, and recall accuracy. In a more rigorous second study, the experimental group showed similarly large improvements, whereas a control group showed no improvement from practicing fraction number line estimates without feedback. The results provide evidence for the effectiveness of interventions emphasizing fraction magnitudes and indicate how psychological theories and research can be used to evaluate specific recommendations of the Common Core State Standards.

## Introduction

Many children and adults struggle with fractions. On one National Assessment of Educational Progress (NAEP), a nationwide test given to a very large, representative sample of U.S. children, only 49% of eighth graders correctly ordered ^2^∕_7_, ^1^∕_2_ and ^5^∕_9_ from least to greatest. On another NAEP, only 55% of 8^th^ graders correctly solved a simple word problem involving fraction division [1, 2]. Despite fraction instruction beginning in elementary school, many people fail to gain a firm understanding of fractions and harbor misconceptions through high school and college [3–6].

This is a serious problem, because understanding of fractions is a foundational mathematical skill. Early fraction knowledge strongly predicts later math achievement [7–9], even after children’s IQ, reading comprehension, working memory, whole number arithmetic knowledge, race, ethnicity, and parental education and income are statistically controlled [7]. Moreover, a sample of 1,000 U.S. Algebra 1 teachers identified a lack of fraction understanding as one of the two largest problems hindering their students’ algebra learning [10].

One major attempt to improve children’s mathematics knowledge in general, and their fraction understanding in particular, is the Common Core State Standards for Mathematics (CCSS-M) [11]. Developed by math teachers, mathematicians, principals, education researchers, and state content experts, the standards have been implemented in 43 states and describe what children should be able to do by the end of each grade. The standards are designed to be more rigorous than most existing state-based standards and to emphasize deeper understanding of fewer topics. For fractions, the standards emphasize that children should understand fractions as numbers with magnitudes that can be compared and ordered, as shown by the cluster headings of “Develop understanding of fractions as numbers” and “Extend understanding of fraction equivalence and ordering” within the section of the document on fractions.

A limitation of the CCSS-M recommendations is that they were not in general based on empirical evidence, but rather on the professional judgments of people with relevant knowledge. This was inevitable, given the very large number of topics that are part of the mathematics curriculum and the lack of well-controlled experimental studies regarding effective teaching techniques for many, probably most, of them. Nonetheless, it seems essential to test key aspects of the recommendations to determine their utility.

While the standards do not dictate how teachers should teach the content in order to reach the standards, they do suggest the type of learning activities that should be emphasized. Within the domain of fractions, one recommendation is that third grade instruction should emphasize accurate placement of fractions on a number line. This differs from the traditional emphasis in U.S. mathematics instruction on circular and rectangular diagrams, where fractions are generally expressed as shaded parts of a whole [12]. The standards’ focus on understanding fractions as numbers with magnitude dovetails with recent emphasis within cognitive psychological theories on the centrality of magnitude understanding to mathematical knowledge.

### Importance of Understanding Numerical Magnitudes

Over the past decade, researchers have identified children’s understanding of numerical magnitudes as a central component of their overall mathematical knowledge. Children’s ability to approximate numerical magnitudes, as measured by their accuracy at placing numbers on a number line, estimating the answers to arithmetic problems and/or estimating the number of objects presented, is strongly related to their math achievement test scores both concurrently [13, 14] and longitudinally [15–17]. In fact, children’s accuracy on a number line task in 1^st^ grade predicts their growth in math achievement through 5^th^ grade, even after controlling for intelligence, working memory, processing speed and other early numerical skills [15].

These relations are causal as well as correlational. Children who participate in interventions designed to improve their understanding of whole number magnitudes show improvements on untrained magnitude tasks [18–20] and on learning novel arithmetic problems [21, 22].

The integrated theory of numerical development [23, 24] suggests that this relation between numerical magnitude understanding and mathematics achievement should hold not just for positive whole numbers, but for all types of numbers. A main tenet of the theory is that a crucial part of mathematical development is understanding that all numbers have magnitudes that can be ordered and compared. Thus, understanding fraction magnitudes is a key step in mathematical development.

As with whole numbers, understanding of fraction magnitudes is related to overall math achievement both concurrently [23, 25] and longitudinally [7–9]. These findings and previous ones with whole numbers suggest that it should be possible to increase children’s fraction magnitude understanding and that the improved understanding should transfer to tasks that were not trained. As described below, current interventions do just that, but are lengthy and multifaceted and do not allow for specification of the components that produce the gains. The present study seeks to provide evidence that a short, simple intervention can also improve fraction magnitude understanding.

### Prior Interventions

Previous studies have shown increases in children’s fraction knowledge following interventions that emphasize fraction magnitude understanding. Moss and Case [26] created a rational numbers curriculum that emphasized connections among percentages, decimals and fractions; comparing and ordering their magnitudes; games, songs, and monetary transactions; and many other activities involving rational numbers. In comparison to children exposed to a traditional curriculum, children who received this experimental curriculum were better able to solve nonstandard problems (e.g., “What is ½ of ^1^∕_8_?”) and compare and order rational numbers, though they were equivalent at solving standard fraction arithmetic problems (e.g., “What is 3 ¼–2 ½?”) [26, 27]. Similarly Saxe, Diakow, and Gearhart [28] showed that a curriculum unit that emphasized placing integers and fractions on number lines was more effective at improving students’ understanding of fraction magnitudes than a well-regarded traditional curriculum. Moreover, Fuchs and colleagues found that at-risk learners who were taught using a curriculum that focused on comparing fractions and placing them on a number line learned more than children taught with a traditional curriculum that described fractions as parts of a whole [29, 30].

These are impressive demonstrations that well-conceived instruction over many weeks, which includes an emphasis on magnitude understanding, can produce large gains in fraction knowledge. However, because the studies were so multifaceted, the source of their effectiveness remains uncertain. To better specify the processes through which interventions designed to improve fraction knowledge exercise their effects, the present study examines learning from a brief intervention tightly focused on fraction magnitudes.

### Current Study

Prior research on children’s understanding of decimal magnitudes showed that playing a brief computer game was effective in improving understanding of decimal magnitudes with both American [31] and German [32] children. In the *Catch the Monster* game, children were presented with a 0–1 number line and a decimal that indicated a monster’s position. Children were encouraged to use the decimal to estimate the monster’s position on the line and received feedback regarding each estimate’s accuracy. If the estimate was sufficiently accurate, the monster died a dramatic death; if not, the monster laughed at and mocked the child.

**Fig 1:**
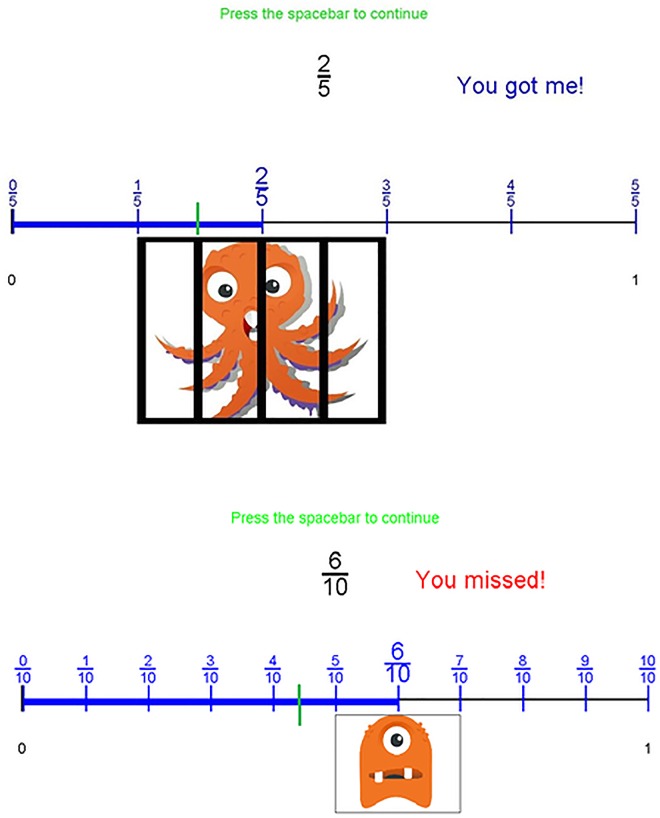
A sample correct trial from the least demanding level of “Catch the Monster with Fractions” (top) and a sample incorrect trial from the most demanding level (bottom).

We adapted this previously successful game to create, *Catch the Monster with Fractions*, which has added features to deal with the more complex concept of fraction magnitudes. *Catch the Monster with Fractions* has three main features. First, in line with the emphasis of the CCSS-M (standard 3.NF.A.1, Table 1) and with children’s limited understanding of fraction notation, we presented a conceptual framework for thinking about the meaning of fractions that emphasized unit fractions (fractions with 1 as the numerator, such as ^1^∕_3_ and ^1^∕_8_). This conceptual framework was presented at the outset of the intervention. It introduced unit fractions, showed children how to divide the number line into the number of segments indicated by the denominator, and pointed out that unit fractions with larger denominators are closer to zero on a number line than unit fractions with smaller denominators. Second, after each estimate of the monster’s position, the child was given detailed feedback, as shown in Fig 1. With the child’s estimate remaining visible, the number line was divided into the number of equal-size segments indicated by the denominator, each segment was labeled with the fraction that expressed the number of segments between it and the origin, and the magnitude of the fraction was emphasized with a bolded line that ran from zero to the presented fraction. This detailed feedback allowed children to see why their answer was correct or incorrect and provided additional opportunities to learn about fraction magnitudes. It also matched the recommendation from the CCSS-M (3.NF.A.2.B) that children should understand that a fraction ^*a*^*∕*_*b*_ on a number line consists of *a* segments of ^1^∕_*b*_ length. Finally, practice was presented in an engaging game setting. Rather than simply learning about fractions, children were trying to capture escaped monsters. This was designed to help children remained motivated even when they were struggling to accurately place fractions on the number line.

**Table 1:** Relevant Common Core State Standards for Mathematics.

Standard	Text
3.NF.A.1	“Understand a fraction ^1^∕_b_ as the quantity formed by 1 part when a whole is partitioned into b equal parts; understand a fraction ^a^∕_b_ as the quantity formed by a parts of size ^1^∕_b_.”
3.NF.A.2	“Understand a fraction as a number on the number line; represent fractions on a number line diagram.”
3.NF.A.2.A	“Represent a fraction ^1^∕_b_ on a number line diagram by defining the interval from 0 to 1 as the whole and partitioning it into b equal parts. Recognize that each part has size ^1^∕_b_ and that the endpoint of the part based at 0 located the number ^1^∕_b_ on the number line.”
3.NF.A.2.B	“Represent a fraction ^a^∕_b_ on a number line diagram by marking off a lengths ^1^∕_b_ from 0. Recognize that the resulting interval has size ^a^∕_b_ and that its endpoint located the number ^a^∕_b_ on the number line.”
4.NF.A.2	“Compare two fractions with different numerators and denominators, e.g., by creating common denominators or numerators, or by comparing to a benchmark fraction such as ½. Recognize that comparisons are valid only when the two fractions refer to the same whole. Record the results of comparisons with symbols >, =, or <, and justify the conclusions, e.g., by using a visual fraction model.”

On the pretest and posttest, children completed three tasks that varied in their distance from the practiced task. In the most similar task, children estimated locations of fractions on number lines; this task differed from the game in the absence of feedback, the lack of a game setting, and the particular fractions presented. Next, a fraction magnitude comparison task required children to decide if a presented fraction was greater than or less than 3/5. This was less similar to the activities in the game, in that it required relative rather than absolute judgments. Magnitude comparison assessed another CCSS-M standard, one that states that children should be able to compare fractions with unequal numerators and denominators (4.NF.A.2). Finally, in the far transfer task, we examined children’s memory for fractions. Prior research has shown that when whole numbers are included in vignettes, the numbers later recalled by children with good magnitude knowledge are closer to the numbers that were presented than are the numbers recalled by children with weaker magnitude knowledge [33]. Using the same reasoning, we expected children with a more precise understanding of fractions to recall fraction magnitudes more accurately, even when they did not remember the exact fractions that were presented. Study 1 was a preliminary study with a simple pretest/posttest design conceived to test if the intervention was effective. Study 2 built on the results of Study 1 to test the intervention against control activities.

## Study 1

### Method

#### Participants

The participants were 26 fourth and fifth graders at two urban charter schools and one suburban public school near Pittsburgh, PA (*M* age = 10.60 years, *SD* = 0.49, 58% 4^th^ grade, 58% female, 69% Black, 19% White, 8% biracial, 4% Asian). Both charter schools served a primarily low-income population, but students at all three schools scored at or above the state average on a standardized test of math achievement.

#### Materials

Full descriptions of the materials, instructions, and procedure are provided in S1 Materials. Here we provide summaries of each.

Catch the monster with fractions. The experimental intervention consisted of brief conceptual instruction, practice in placing fractions on a number line, and feedback immediately after each estimate. First, the children received approximately 3 minutes of conceptual instruction about unit fractions. The instruction defined unit fractions as fractions in which the numerator is 1, and it then provided several illustrations. Next, children were shown how to locate unit fractions on a 0–1 number line, and shown that unit fractions with larger denominators correspond to smaller segments of the number line. Finally, children were told that the numerator in the fraction indicated the number of segments of the unit fraction (e.g., ^3^∕_4_ is 3 of the ^1^∕_4_ segments). During the instruction, visuals were presented on the computer screen while the experimenter read the script and pointed at the relevant part of the display.

Next, children played *Catch the Monster with Fractions*. They were told that the monsters had escaped and that their knowledge of fractions was needed to recapture them. On each trial, children saw a number line with “0” on the left end, “1” on the right end, and a fraction above the line that indicated the monster’s hiding place. Children were instructed to catch the monster by clicking the computer’s mouse at the number line location where the fraction belongs.

After children clicked on the number line, they simultaneously saw the location that they clicked, the correct location of the fraction, and whether their estimate was close enough to catch the monster (Fig 1). In addition, the number line was segmented into the number of labeled parts indicated by the denominator, and the line was bolded from 0 to the location on the number line of the presented fraction. After viewing the feedback for two seconds, children could move onto the next trial. As children improved at locating the fractions, the monster got smaller and required more precise estimates of the fractions’ locations. At the start of the game, estimates had to be within 20% of the monster’s midpoint to catch it. Thus, as shown in the top panel of Fig 1, when the fraction presented was ^2^∕_5_, the monster would be caught by estimates ranging from ^1^∕_5_ to ^3^∕_5_. If the child correctly answered five consecutive trials at that tolerance, a smaller monster was presented, and the estimates had to be within 15% of its midpoint. When children answered five consecutive trials correctly, they moved to the final level, where estimates had to be within 10% of the midpoint (bottom panel, Fig 1). Children played the game for ten minutes or until they answered five consecutive problems correctly at the most demanding level.

To-be-estimated fractions were drawn from the set of 45 fractions with integer numerators and denominators less than or equal to ten and magnitudes between 0 and 1, exclusive. On each trial, a fraction was randomly drawn from the set without replacement. Because fractions that met these specifications tended to have large denominators (1 fraction with a denominator of 2 but 8 with a denominator of 9), once a denominator was presented three times, no more fractions with that denominator were shown until the entire set was exhausted. Then, the set was reshuffled, and the denominator limits were reset. This helped ensure that children received practice with smaller as well as larger denominators.

Pre- and posttest measures of fraction knowledge. Number line estimation: Children were presented 12 0–1 number lines. Above each line was a to-be-estimated fraction. Children made their estimates by moving the cursor to the desired position and clicking the mouse button. Two sets of fractions served as the pretest and posttest. Each child saw one set on the pretest and the other on the posttest, counterbalanced across participants. Each set included three fractions from each quarter of the number line; no denominator occurred more than three times in a set.

Magnitude comparison: On each trial, children were shown a 0–1 number line with the location of ^3^∕_5_ marked and asked whether each of 15 fractions was less than or greater than ^3^∕_5_. One set of fractions was presented at pretest and another at posttest, counterbalanced across participants. In each set, eight fractions were smaller than ^3^∕_5_ and seven were larger. Denominators ranged from three to ten; no denominator appeared more than three times in a set.

Recall: On the recall task, children were read short vignettes and asked to remember information from the story. Children were instructed to pay attention to the specifics of the story, because they would be questioned about what they heard. Each vignette contained two fractions and some filler information. Sample vignettes and questions are shown in Table 2.

**Table 2:** Sample Items from the Fraction Recall Task.

Story	Question 1	Question 2	Question 3
Jamie ordered a pepperoni pizza for her and her friends. Jamie ate ^4^∕_7_ of the pizza and her friend Sam ate ^2^∕_9_ of the pizza.	What kind of pizza did Jamie order?	What fraction of the pizza did Jamie eat?	What fraction of the pizza did Sam eat?
Sarah wanted to find something to read at her school’s library. Sarah’s favorite books are about horses. On one shelf, she saw that ^3^∕_7_ of the books were fiction and on another shelf ^2^∕_5_ of the books were nonfiction.	What fraction of the books were fiction?	What fraction of the books were nonfiction?	What are Sarah’s favorite books about?
Mr. Smith asked the children in his class how they liked to travel best. ^5^∕_8_ of the children in his class liked airplanes best and ^3^∕_10_ of the children in his class liked cars best. Mr. Smith likes to travel by train.	How does Mr. Smith like to travel?	What fraction of the children liked airplanes best?	What fraction of the children liked cars best?

After the experimenter read the story aloud, the child was asked to count backwards by threes from a two-digit number for 15 seconds. Then, three questions about the story were presented. Children were asked to recall both fractions and one other detail about the story. Six vignettes were presented as the pretest and another six as the posttest, counterbalanced across participants.

### Procedure

All children participated individually in a quiet room at their school during the school day. Pretest, intervention, and posttest together took approximately 30 minutes and occurred during the same session. Children first completed the fraction recall, number line estimation, and magnitude comparison pretests, in that order. They then received the conceptual instruction and played *Catch the Monster with Fractions*. Finally, they completed the posttest tasks in the same order as on the pretest. Approval for the study was provided by the Carnegie Mellon University’s Institutional Review Board, and written consent was obtained from both the parent and child.

### Results

#### Performance on *Catch the Monster with Fractions*

Children completed a median of 30 trials while playing the computer game, and their estimates were marked as incorrect on an average of 25% (*SD* = 16) of trials. Most participants (16 of 26, 61%) successfully completed all three levels after an average of 5.5 minutes (*SD* = 1.5) and 27 trials (*SD* = 9). For the purpose of comparison, the minimum number of trials required to complete the game was 15. Among the other ten children, five reached, but did not complete, level three, three reached level two, and two did not pass level one.

#### Fraction number line estimation

Estimation accuracy was measured using percent absolute error (PAE), defined as: PAE = (|Participant’s Answer–Correct Answer|)/ Numerical Range X 100. For example, if a participant was asked to locate ^1^∕_5_ on a 0–1 number line, and marked the location corresponding to ^1^∕_4_; PAE would be 5% ((|0.25–0.20|)/1 X 100). PAE varies inversely with accuracy; the higher the PAE, the less accurate the estimate.

Estimates improved greatly from pretest to posttest, *t*(25) = -4.73, *p* < .001, *d* = -1.10. Pretest PAE averaged 19% (*SD* = 11), posttest PAE averaged 10% (*SD* = 6).

#### Magnitude comparison

Accuracy on the magnitude comparison task also improved, from 62% correct (*SD* = 19) on the pretest to 70% (*SD* = 19) on the posttest, *t*(25) = 2.37, *p* = .026, *d* = 0.48.

#### Recall

If the intervention improved fraction magnitude understanding and that knowledge influenced encoding and retrieval, recall of fractions in the vignettes should be closer to the original values on the posttest than on the pretest. We used PAE as our measure of recall accuracy. While all to-be-remembered fractions were less than one, children occasionally recalled fractions above one, which could lead to a PAE > 100%. PAEs on those 2% of trials were trimmed to 100%.

As expected, pretest accuracy on the memory task was related to pretest number line estimation PAE, *r*(24) = .54, *p* = .004, and magnitude comparison accuracy, *r*(24) = -.47, *p* = .014, demonstrating that fraction knowledge was related to recall accuracy (the negative correlation was due to higher magnitude comparison accuracy and lower PAE reflecting better knowledge). This replicates the findings of Thompson and Siegler [33] and extends their findings from whole numbers to fractions.

Especially striking, recall accuracy of the fractions in the vignettes improved after playing *Catch the Monster with Fractions*. PAE decreased from 20% (*SD* = 12) on the pretest to 16% (*SD* = 9) on the posttest, *t*(25) = -2.47, *p* = .021, *d* = -0.52. Recall of the exact fractions in the vignettes did not increase from pretest to posttest, 3.12 vs. 3.15, *t* < 1, nor did correct recall of filler information, 3.77 vs. 3.65, *t* < 1. These findings provided discriminant validity for the interpretation that improved recall accuracy reflected improved knowledge of fraction magnitudes, rather than improved memory for exact numerical information or improved facility with the types of questions asked.

### Discussion

Performance on all three outcome measures improved from pretest to posttest. Thus, the intervention showed promise for improving students’ fraction understanding. However, Study 1 utilized a simple pretest/posttest design, so the increases could have been due simply to increased familiarity with the outcome measures, experimental situation, or some other third variable. Therefore, to replicate the initial findings and test the effectiveness of the intervention more rigorously, in Study 2 we randomly assigned children to experimental and control groups. Participants in the control group practiced placing fractions on a number line for the same number of trials as participants in Study 1, but did not receive feedback or instruction, whereas the experimental group received the same instruction as in Study 1. This control group was designed specifically to examine if the gains seen in the first study were due to additional exposure to fractions and fraction number line estimation or if the gains were due to some combination of the instruction, game and feedback.

## Study 2

### Method

#### Participants

Participants were 51 fifth graders (*M* age = 10.73, *SD* = 0.39, 61% female, 69% White, 20% Black, 8% biracial, 2% Asian, 2% Hispanic), who came from three schools near Pittsburgh, PA: an urban charter school, a private Catholic school, and a suburban public school. Students at all three schools scored at or above the state average on a standardized test of math achievement. Of the 51 children, 25 practiced placing fractions on number lines and 26 played *Catch the Monster with Fractions*. Due to a computer malfunction, one participant was excluded from the control condition on the fraction magnitude comparison task.

#### Control activities

Children in the control condition of Study 2 placed fractions on a 0–1 number line for 30 trials without feedback. The computer program that presented problems and recorded responses was the same as in Study 1 and in the experimental group of Study 2. The 30 trials matched the median number of trials during *Catch the Monster with Fractions* in Study 1; it was somewhat higher than the median of 24 trials for the experimental group in Study 2. The fractions’ denominators ranged from 2–10, as in the experimental condition.

#### Procedure

Participants were randomly assigned to either the control or experimental condition. The experimental group received the same intervention as in Study 1 featuring both the conceptual instruction and playing *Catch the Monster with Fractions*. Participants were again tested individually in a quiet space in their school. The three pretest and posttest measures were unchanged from Study 1.

### Results

#### Performance on *Catch the Monster with Fractions*

Of the 26 children who received the intervention, 18 (69%) met the criterion at the highest level after an average of 5.1 minutes (*SD* = 1.5) and 24 trials (*SD* = 10). Of the eight children who timed out without meeting that criterion, four reached but did not complete level three, three reached but did not complete level two, and one did not complete level one. Across all children who received the intervention, the median number of trials received was 24; among the estimates, 21% (*SD* = 17) were marked as incorrect (too far from the correct location of the fraction).

#### Fraction number line estimation

**Fig 2:**
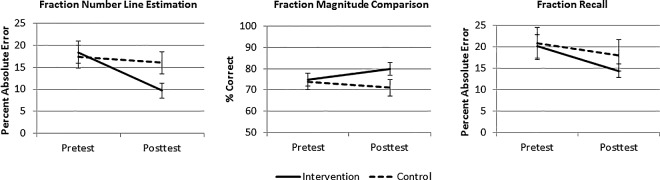
Mean pretest and posttest performance on fraction number line estimation, magnitude comparison, and recall for children in the control and experimental conditions (Study 2).

A 2 (Condition: experimental or control) X 2 (Time of test: pretest or posttest) ANOVA revealed an interaction between condition and time of test, *F*(1, 49) = 10.42, *p* = .002, η^2^_p_ = .18. As shown in the leftmost panel of Fig 2, children who received the experimental intervention were much more accurate at placing fractions on the number line on the posttest (PAE = 10%, *SD* = 9) as compared to the pretest (PAE = 18%, *SD* = 13), *t*(25) = -4.14, *p* < .001, *d* = -0.86. In contrast, estimation accuracy of children in the control group did not change from pretest (PAE = 17% *SD* = 13) to posttest (PAE = 16%, *SD* = 13), *t*(24) = -1.54, *p* = .136, *d* = -0.31.

#### Fraction magnitude comparison

A parallel ANOVA indicated that condition and time of test also interacted on the fraction magnitude comparison task, *F*(1, 48) = 4.32, *p* = .043, η^2^_p_ = .08. As shown in the middle panel of Fig 2, playing the game tended to improve children’s magnitude comparison accuracy from pretest (75% correct, *SD* = 18) to posttest (80% correct, *SD* = 15), *t*(25) = 1.87, *p* = .074, *d* = 0.35. Children in the control group again showed no pretest-posttest improvement (74% correct, *SD* = 20; 71% correct, *SD* = 22), *t*(24) = 1.13, *p* = .272, *d* = 0.23.

#### Fraction recall

As in Study 1, PAEs > 100% were trimmed to 100% (4% of trials). Pretest fraction recall PAE was again correlated with both number line estimation PAE, *r*(49) = .29, *p* = .038, and magnitude comparison accuracy, *r*(49) = -.32, *p* = .023.

On this measure, time of test and condition did not interact (*F* < 1; rightmost panel of Fig 2). As in Study 1, recall of children who received the intervention improved from pretest (PAE = 20%, *SD* = 14) to posttest (PAE = 14%, *SD* = 8), *t*(25) = -2.28, *p* = .031, *d* = -0.49. Unexpectedly, however, children who practiced fraction number line estimation without feedback also improved, though the improvement was smaller and marginally significant (pretest PAE = 21%, *SD* = 19, posttest PAE = 18%, *SD* = 18), *t*(24) = -1.80, *p* = .085, *d* = -0.36.

As in Study 1, there was no change from pretest to posttest in recall of the exact fractions, 3.55 vs. 3.55, *t* < 1, or of the filler information, 3.94 vs. 4.29, *t*(50) = 1.48, *p* = .146, *d* = 0.21, suggesting that the increased accuracy of recall was specific to the magnitude information.

#### Existing Knowledge and Acquisition of New Knowledge

We also examined the amount learned from the game among children who had above or below average initial knowledge of fractions. For this analysis we used a combined sample of all the children who played *Catch the Monster with Fractions* in Studies 1 and 2. We first checked whether the 4^th^ and 5^th^ graders showed equal gains from playing the game. Children at both grade levels showed similar gains from pretest to posttest on number line estimation (4^th^ 6.33, 5^th^ 9.38, *t*(50) = 1.02, *p* = .313), magnitude comparison (4^th^ 7.56, 5^th^ 6.13, *t* < 1) and fraction memory (4^th^ 4.15, 5^th^ 5.25, *t* < 1).

We then standardized children’s performance on the three pretest measures and added the three z-scores together to create a composite measure of initial fraction knowledge (number line estimation PAE and fraction memory PAE were reverse scored so that higher scores indicated more knowledge for all three tasks). This composite measure was not correlated with grade level, *r*(50) = .09, *p* = .515.

Students with less initial knowledge showed greater improvements than peers with more initial knowledge. On each task, there was a significant negative correlation between initial fraction knowledge and gains from pretest to posttest; number line estimation *r*(50) = -.48, p < .001; magnitude comparison *r*(50) = -.30, p = .033; fraction memory *r*(50) = -.39, p = .004. At minimum, this shows that the game was effective with students who started with less knowledge. At maximum, it suggests that the game is especially effective with such students. Relevant descriptive statistics are shown in Table 3.

**Table 3:** Descriptive Statistics of Pretest Fraction Knowledge and Gains from Pretest to Posttest for Children who played the Catch the Monster Game.

	*M*	*SD*	Range
Pretest fraction knowledge (z-score)	0	2.31	-5.57–3.61
NLE Gain (PAE)	8.50	9.78	-9.80–39.35
Mag Comp Gain (% correct)	6.54	15.54	-27.67–53.33
Fraction Memory Gain (PAE)	4.93	10.78	-11.80–37.36

Although 18 children timed out of the game without completing Level 3 (the highest level), these children’s fraction knowledge improved from pretest to posttest on both number line estimation and fraction recall: number line estimation pretest PAE 28% (*SD* = 12), posttest 17% (*SD* = 9), *t*(17) = -3.78, *p* = .001, *d* = - 0.91; fraction recall pretest PAE 26% (*SD* = 18), posttest 18% (*SD* = 10), *t*(17) = -2.74, *p* = .014, *d* = -0.83. Magnitude comparison accuracy of these children did not change, pretest 59% correct (*SD* = 16), posttest 63% correct (*SD* = 15), *t*(17) = 1.00, *p* = .331, *d* = 0.26.

## General Discussion

Across two studies, children who received conceptual instruction about fractions that was in accord with the Common Core State Standards and then played *Catch the Monster with Fractions* showed large improvements in fraction magnitude understanding. They more accurately placed fractions on a number line, compared fraction magnitudes, and remembered the magnitudes of fractions in stories on the posttest than the pretest. These gains were consistently found across both studies. In contrast, children who practiced placing fractions on number lines without feedback did not show significant improvements on any of the three tasks.

### Amount and Breadth of Learning

The knowledge gains of children who received the intervention were quite large. The 4^th^ and 5^th^ graders in these studies finished the intervention with average PAEs on the fraction estimation task that were similar to those of 8^th^ graders from Siegler, Thompson and Schneider [23] and more accurate than those of 8^th^ graders in Siegler and Pyke [25]. Yet, children in the present study began with PAEs of 18% and 19%, slightly worse than the 15% observed for 6^th^ graders in Siegler, Thompson and Schneider [23] and similar to the 18% in Siegler and Pyke [25]. Thus, after the present 15-minute intervention, children were as accurate as peers with several years of additional schooling.

Moreover, the intervention was at least as effective for children who started with less knowledge as for ones who started with more. Rather than finding the typical “Matthew effect” where high-knowledge children learn more than low-knowledge children [34, 35], children who started with lower knowledge showed greater improvement. Even children who were unable to finish *Catch the Monster with Fractions* in the allotted time showed improvement in fraction estimation accuracy and memory for fractions.

These results suggest that classroom activities focused on inculcating understanding of unit fractions and locations of fractions on number lines (activities emphasized in the CCSS-M) are likely to improve students’ understanding of fraction magnitudes. However, such activities must also include well-designed feedback. Practice in placing fractions on number lines is insufficient to improve student knowledge. Future research should examine the effectiveness of the intervention when done in whole-class situations and how to expand students’ fraction understanding to include all fractions, rather than simply the proper fractions (those from 0–1) used in the present studies.

In addition to demonstrating the value of number line based fraction activities, these results provide new theoretical evidence for the connection among different types of fraction magnitude knowledge. The intervention, which dealt specifically with placing fractions on a number line, produced gains not only on number line estimation but also on fraction magnitude comparison and memory for fractions. Thus, the students were not solely gaining procedural knowledge of how to place fractions on number lines, but rather emerged with a better understanding of fractions more generally. The results from the memory task are particularly striking in how different the task was from the task trained during the intervention. This far transfer provides further evidence for the assumption of the integrated theory of numerical development [23, 24] that understanding numerical magnitudes is crucial for a wide range of mathematical knowledge.

### Limitations and Future Directions

Several limitations of the present investigation should be noted. In both studies, the posttest was presented immediately after the intervention. Thus, one clear avenue for future research is to investigate the persistence of the benefits. Does the intervention change the way that children understand fractions in the long-term, or are booster sessions necessary to consolidate the change? In addition, we purposefully developed the *Catch the Monster with Fractions* game to include three elements that we hypothesized to be beneficial for learning: unit fractions instruction, elaborative feedback, and an engaging game context. Now that it has been established that the intervention has relatively broad, positive effects, future research is needed to determine which of these features are crucial for learning. Finally, our sample was ethnically diverse, but the students attended schools with average or above average math achievement. Establishing the effectiveness of the intervention with students at schools with lower mathematics achievement is thus another important goal. The greater learning of children with less pretest knowledge suggests that the present approach will be effective at such schools, but that remains to be demonstrated. Finally, the ideal timing of the intervention remains to be established. We hypothesize that the intervention is particularly effective when students have some but not great knowledge of fractions (as was true for most students in the present studies), but again, the ideal timing of the intervention is currently unknown.

### Testing and Improving the Common Core State Standards

More generally, the present studies illustrate a method that seems applicable to testing the common core state standards in their current form and developing research-based modifications where necessary. The present results support the standards’ suggestion that understanding unit fractions and learning to place fractions on number lines are key capabilities that help deepen children’s understanding of fractions. However, the present results do not suggest that all of the standards are equally effective; whether that is the case remains to be seen. Experimentally testing the standards’ recommendations can help make them increasingly evidence-based. It is also important to note that our findings do not mean that other types of fraction instruction are not effective. We found positive results after emphasizing unit fractions and placing fractions on number lines, but that does not mean that other instructional techniques would not show similar gains. However, given students’ current struggles with fractions, as documented in the introduction, we would suggest that the typical modes of instruction are not serving the current needs of students.

This approach of testing the effects of implementing recommendations from the standards is entirely in keeping with the spirit with which the standards were proposed. The authors of the standards explicitly stated, “One promise of common state standards is that over time they will allow research on learning progressions to inform and improve the design of standards to a much greater extent than is possible today” [11]. We encourage other investigators to test additional specific recommendations of the standards, so that the research community can produce the scientific evidence necessary for informed debate and improvements in this major educational initiative.

## Supporting Information

S1 Data Data from Study 1. (SAV)

S2 Data Data from Study 2. (SAV)

S1 Materials Full descriptions of the materials, instructions, and procedure for both the intervention and the pretest/posttest measure. (DOCX)
