# Supplementary material for: Improving Children’s Knowledge of Fraction Magnitudes
Source: PLoS One. 2016 Oct 21;11(10):e0165243. doi: 10.1371/journal.pone.0165243 (PMC5074569; doi:10.1371/journal.pone.0165243)
Supplement: S1 Materials — (DOCX) [file pone.0165243.s003.docx]

**Unit fractions instruction**

A unit fraction is a fraction in which the numerator is 1. Examples of unit fractions include ¼, ½, 1/15, 1/19, 1/56 and 1/3. Can you give me an example of a unit fraction? (wait for example)


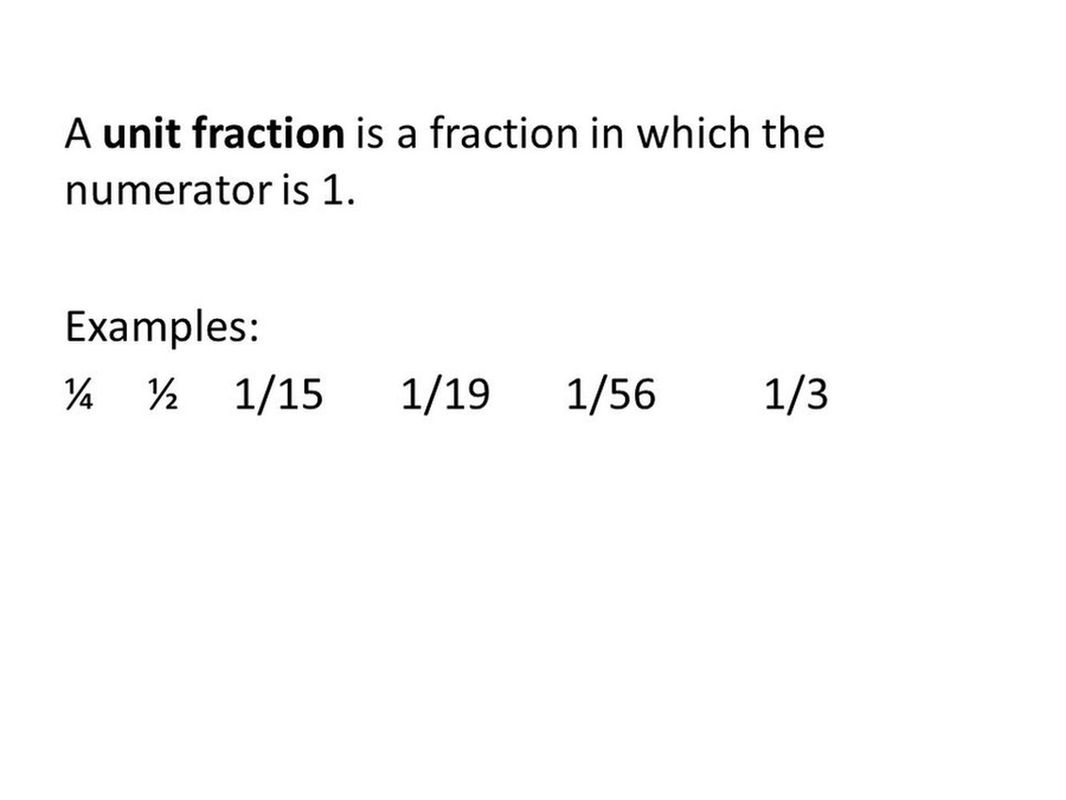


One way to figure out what the size of a unit fraction is for any fraction is to imagine a number line with 0 at one end and 1 at the other (show number line). Then, you divide the 0-1 number line into the number of segments indicated by the denominator.


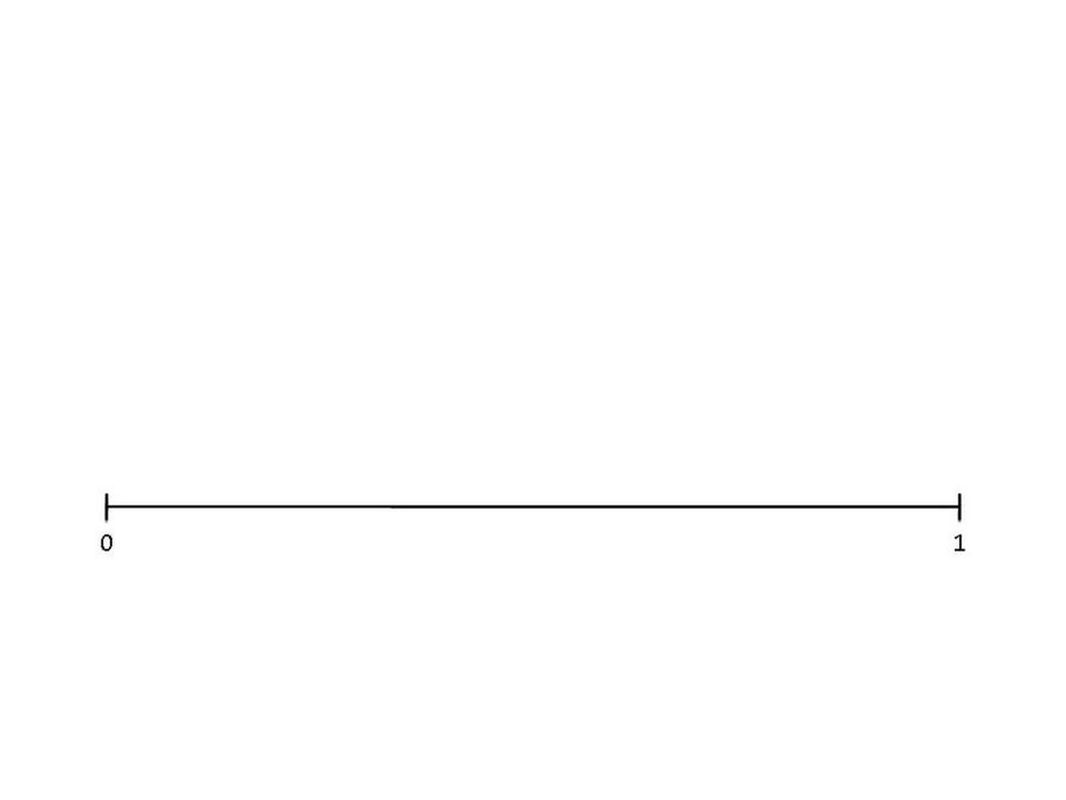


So, if the fraction is 1/3, you imagine dividing the number line into three equal segments (show the number line below divided into three segments). The unit fraction is one of the segments.


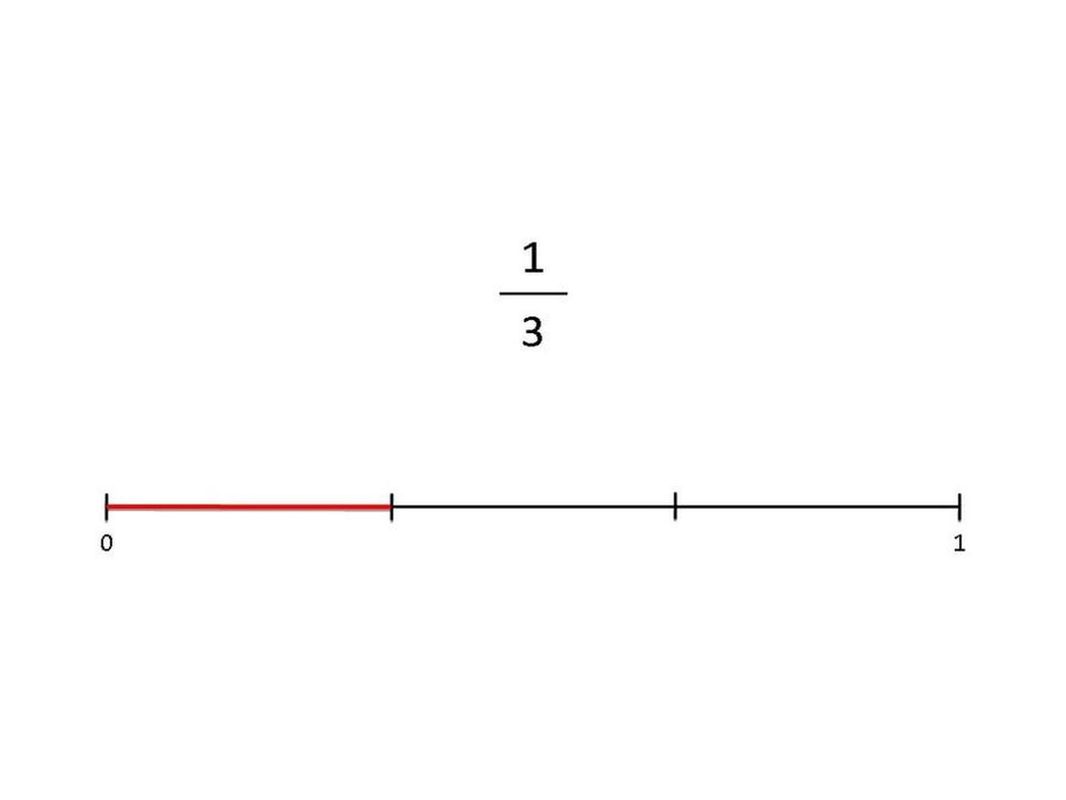


If the fraction is ¼, the denominator is 4, so you divide the number line into 4 equal segments, and the unit fraction is one of these segments (show number line below divided into four segments).


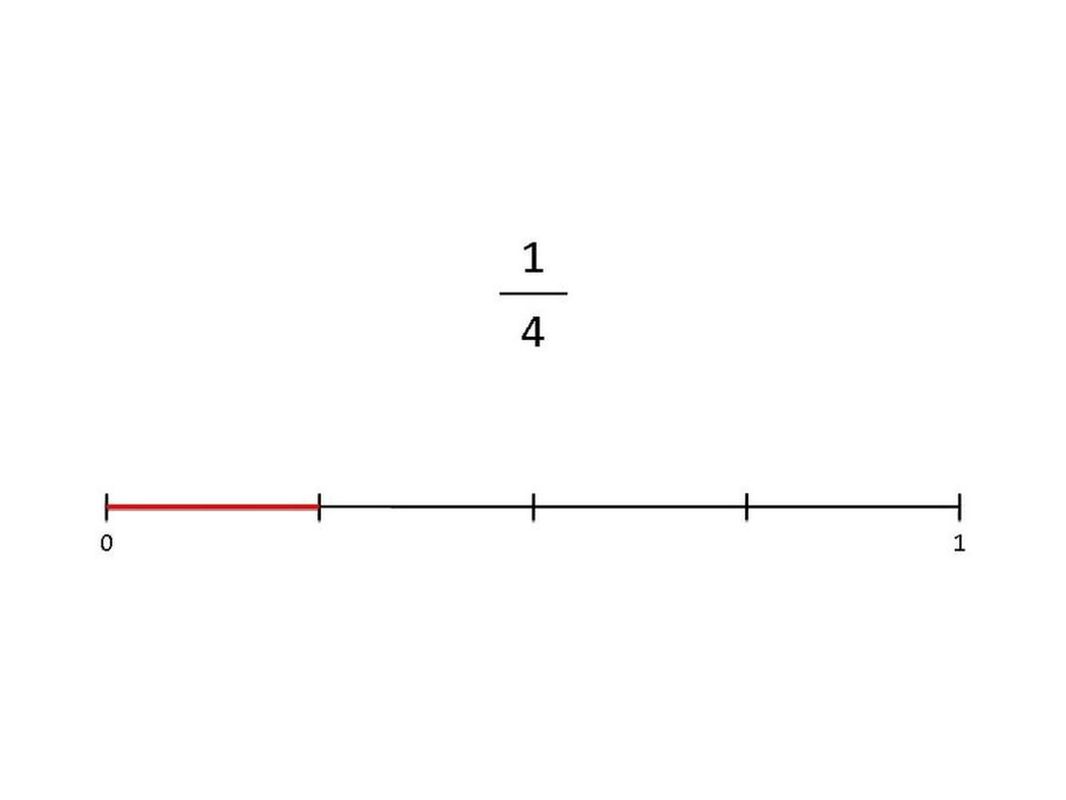


If the fraction is 1/8, the denominator is 8, so you divide the line into eight equal segments and the unit fraction is one of them (show number line below divided into eight segments).


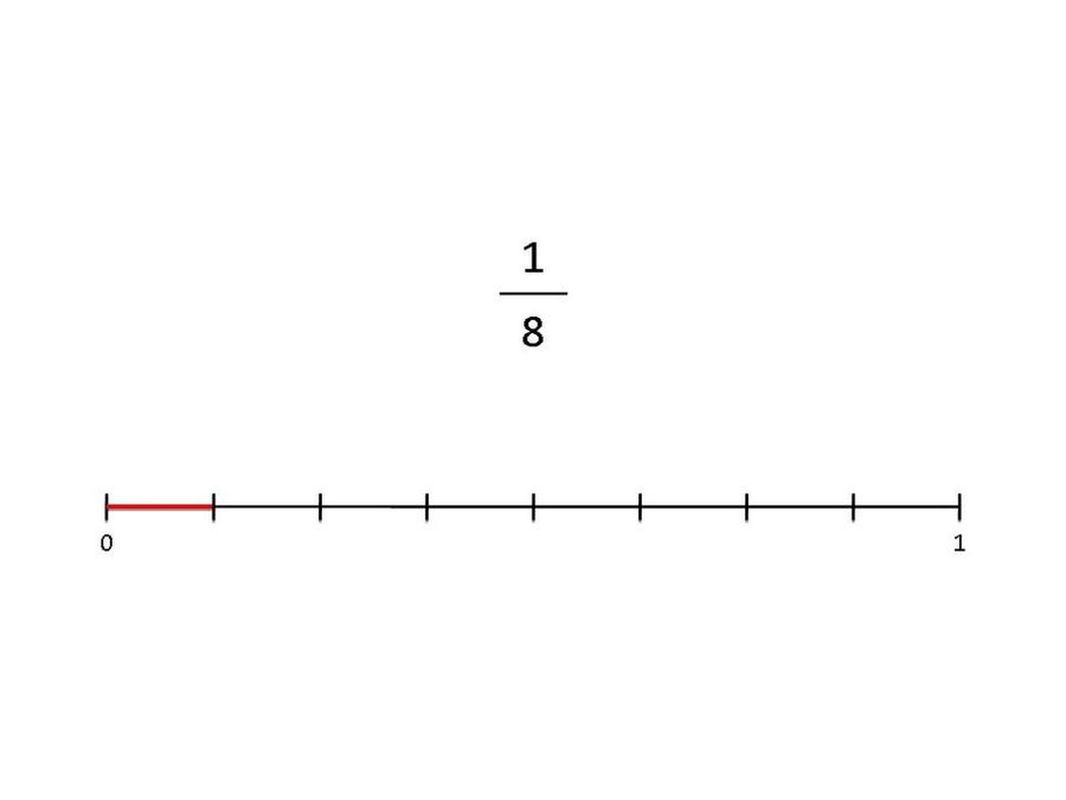


As you can see, the bigger the denominator is, the smaller each segment is (point to denominator of 1/3, ¼, 1/8 and colored segments while saying text below). With 1/3, each segment is this big; with ¼, it’s a little smaller, with 1/8 it’s even smaller. That’s because when you divide the same whole into more parts, each part must be smaller. So that’s what the denominator indicates – the number of segments into which the whole is divided.


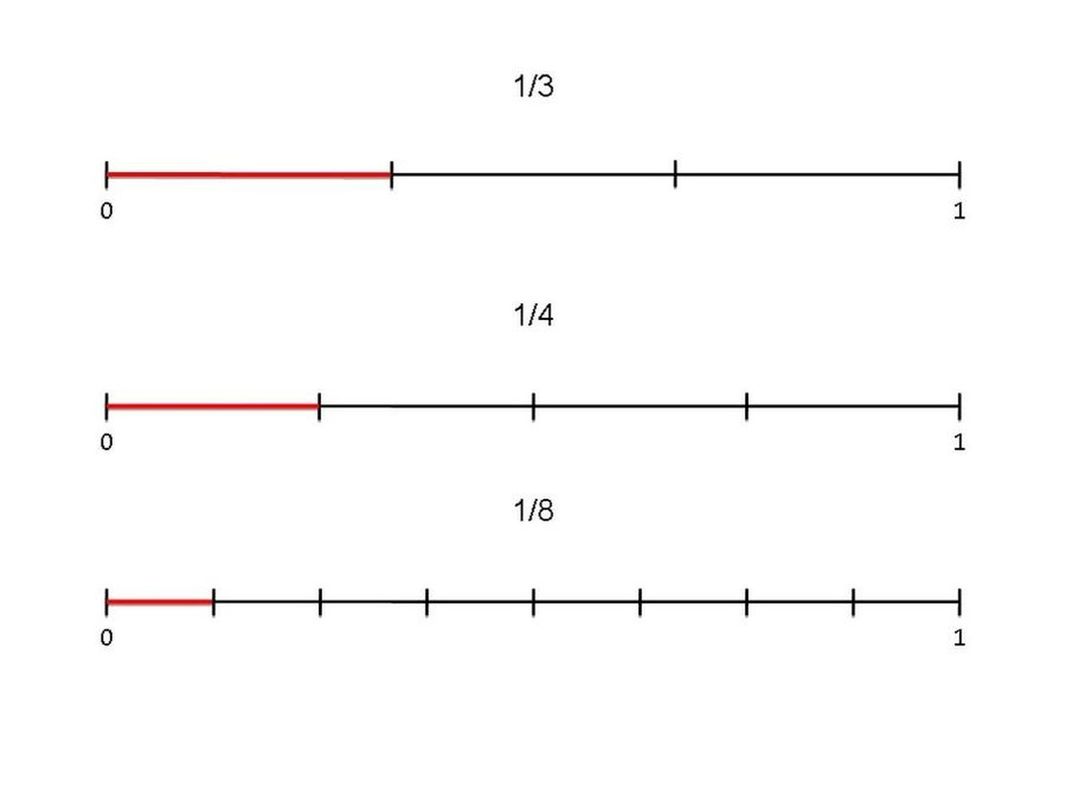


The numerator in a fraction indicates the number of unit fractions in the fraction that you’re working with. When the numerator is one, for example with ¼, the fraction equals one of the ¼ segments whose size was indicated by the denominator (show the highlighted ¼ segment). If the numerator is 3, as with ¾, the fraction equals three of the ¼ segments (show the three ¼ segments).


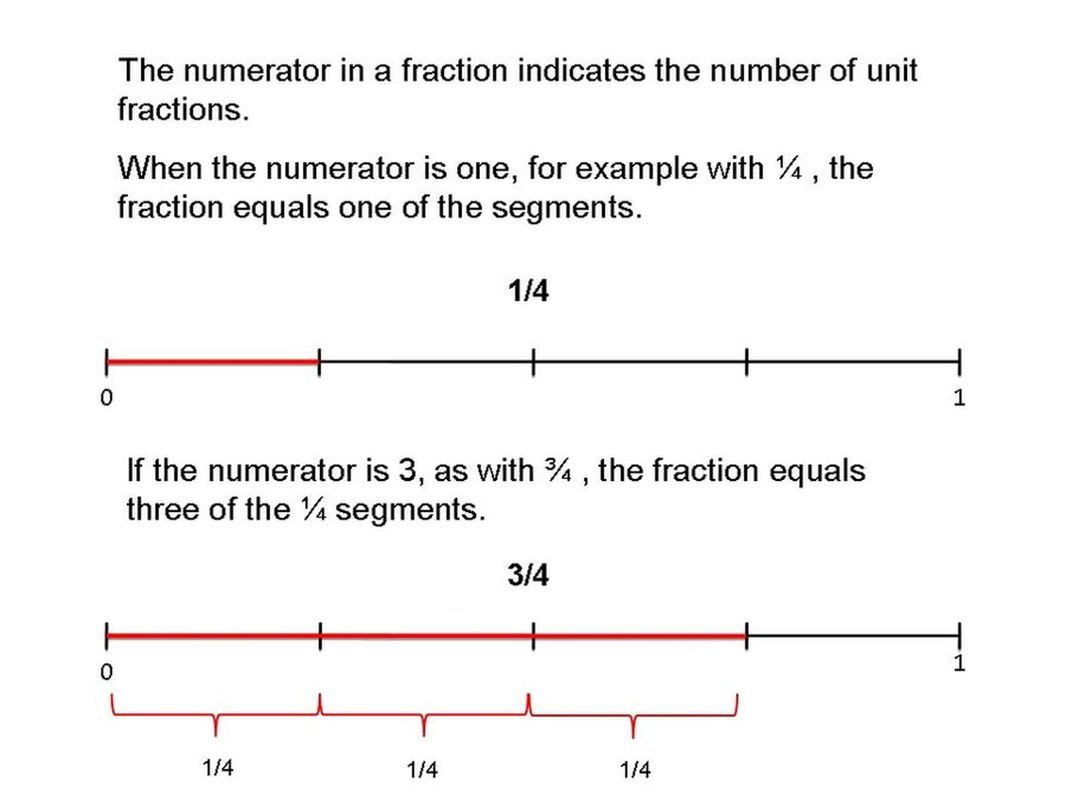


**Catch the Monster instructions**

The monsters have escaped!! Use your knowledge of fractions to help recapture the monsters. The monsters are hiding somewhere on this number line. The line goes from 0 on the left to 1 on the right. A fraction will appear above the line to show where the monster is hiding. Your job is to decide where the fraction belongs on the number line to try and catch the monster. If the fraction’s size is closer to 0, your mark should be closer to 0. If the fraction’s size is closer to 1, your mark should be closer to 1. You will use the mouse to click on the line to try and catch the monster. Ready? Let’s catch some monsters!!

**Number line estimation instructions**

For this task, you will estimate the size of some fractions. Here’s a number line going from 0 to 1. You will see a fraction above the line and your job is to decide where the fraction belongs on the number line. If the fraction’s size is closer to 0, your mark should be closer to 0. If the fraction’s size is closer to 1, your mark should be closer to 1. You will use the mouse to click on the line to show me where you think it goes. Placing the fractions on the number line may help you learn its size, which will help you on the other fraction tasks. Any questions?”

**Number line estimation stimuli**

Set A: 1/7, 1/5, 2/9, 2/8, 3/10, 4/10, 4/8, 3/5, 2/3, 6/8, 7/9, 6/7

Set B: 1/9, 1/6, 2/10, 2/7, 2/6, 2/5, 3/6, 5/9, 6/10, ¾, 4/5, 7/8

**Fraction magnitude comparison instructions**

For this task you will be comparing a fraction’s size to 3/5. You will see a number line going from 0 to 1 showing you how big 3/5 is. Under the number line, you will see a fraction. Your job is to decide if the fraction is less than or greater than 3/5. If the fraction is less than 3/5 (point to the left of 3/5) press the key marked with the “L”. If the fraction is greater than 3/5(point to the right of 3/5), press the key marked with the “G”. You may want to think about the number line estimation task when you are trying to decide if the fraction is less than or greater than 3/5. When you’re ready to start press the spacebar

**Fraction magnitude comparison stimuli**

Set A: 1/7, 2/9, 3/8, 4/10, 3/7, 4/8, 5/9, 4/7, 5/8, 2/3, 4/5, 5/6, 8/9, 9/10, 5/5

Set B: 1/6, 2/10, 2/7, 3/10, 2/5, 4/9, 4/8, 3/6, 6/9, 5/7, ¾, 7/9, 6/7, 7/8, 6/6

**Fraction recall instructions**

For this task, I am going to read you a story and then ask you some questions afterward. You simply need to recall the information from the story exactly as you heard it. Make sure that you are paying attention to the details of the story so you are able to answer the questions. Any questions?”

**Fraction recall stimuli**

Version A:

1. Sarah wanted to find something to read at her school’s library. Sarah’s favorite books are about horses. On one shelf, she saw that 3/7 of the books were fiction and on another shelf 2/5 of the books were nonfiction.

*Count backwards from 66 by 3s*

Questions:

- What fraction of the books were fiction?
- What fraction of the books were nonfiction?
- What are Sarah’s favorite books about?

1. Mr. Smith asked the children in his class how they liked to travel best. 5/8 of the children in his class liked airplanes best and 3/10 of the children in his class liked cars best. Mr. Smith likes to travel by train.

*Count backwards from 56 by 3s*

Questions:

- How does Mr. Smith like to travel?
- What fraction of the children liked airplanes best?
- What fraction of the children liked cars best?

1. Jamie ordered a pepperoni pizza for her and her friends. Jamie ate 4/7 of the pizza and her friend Sam ate 2/9 of the pizza.

*Count backwards from 76 by 3s*

Questions:

- What kind of pizza did Jamie order?
- What fraction of the pizza did Jamie eat?
- What fraction of the pizza did Sam eat?

1. A farmer who lives in Iowa planted different kinds of vegetables in his garden. 3/8 of the vegetables planted by the farmer were carrots and 1/9 of the vegetables planted by the farmer were potatoes.

*Count back from 53 by 3s*

Questions:

- What fraction of the vegetables planted were carrots?
- What fraction of the vegetables planted were potatoes?
- Where does the farmer live?

1. Mr. Costa asked the children in his class which sport they liked best. 3/7 of children liked football best and 4/10 of children liked the soccer best. Mr. Costa’s favorite is baseball.

*Count back from 98 by 3s*

Questions:

- What is Mr. Costa’s favorite sport?
- What fraction of the children liked football best?
- What fraction of the children liked soccer best?

1. Colleen washes the silverware at an Italian restaurant. 5/10 of the silverware she washes are forks and 2/8 of the silverware she washes are spoons.

*Count back from 49 by 3s*

Questions:

- What fraction of the silverware washed were forks?
- What fraction of the silverware washed were spoons?
- What kind of restaurant does Colleen work at?

Version B:

1. Mrs. Conway asked the children in her class about their favorite foods. 3/8 of the children liked spaghetti best and 2/5 of the children liked pizza best. The other children liked chicken nuggets.

*Count back from 30 by 3s*

Questions:

- What food did the other children like best?
- What fraction of the children liked spaghetti best?
- What fraction of the children liked pizza best?

1. Michael has a bag of M&Ms. 5/8 of the M&Ms were blue and 2/7 of the M&Ms were red. Michael’s favorites are the green M&Ms.

*Count back from 97 by 3s*

Questions:

- What fraction of the M&Ms were blue?
- What fraction of the M&Ms were red?
- What’s Michael’s favorite color of M&M?

1. Jasmine was doing her yard work on Saturday. Jasmine weeded 5/9 of her garden in the morning and 2/10 of her garden in the afternoon.

*Count back from 57 by 3s*

Questions:

- What day was Jasmine doing her yard work?
- What fraction of the garden did Jasmine weed in the morning?
- What fraction of the garden did Jasmine weed in the afternoon?

1. Greg made a pan of chocolate chip brownies. Greg ate 2/6 of the pan of brownies and John ate 3/7 of the pan of brownies.

*Count back from 69 by 3s*

Questions:

- What fraction of the pan of brownies did Greg eat?
- What fraction of the pan of brownies did John eat?
- What kind of brownies did Greg make?

1. Michael did a survey in his class to find out his classmates’ favorite season. 1/5 of students chose Spring and 4/8 of students chose Fall. Michael’s favorite season is Summer.

*Count back from 46 by 3s*

Questions:

- What’s Michael’s favorite season?
- What fraction of the students liked Spring best?
- What fraction of the students liked Fall best?

1. Mr. Jones asked his science class what type of dessert they would like at their class party. 4/10 of students wanted cake and 3/8 of students wanted ice cream.

*Count back from 63 by 3s*

Questions:

- What fraction of the students wanted cake?
- What fraction of the students wanted ice cream?
- What type of class does Mr. Jones teach?

**Number line estimation practice stimuli – control condition Study 2**

1/2, 1/3, 2/3, 2/4, 3/4, 1/5, 3/5, 4/5, 4/6, 5/6, 2/7, 4/7, 5/7, 6/7, 2/8, 4/8, 5/8, 7/8, 1/9, 2/9, 3/9, 5/9, 6/9, 7/9, 1/10, 2/10, 3/10, 4/10, 5/10, 6/10
